# Rapid Ebola tests hold promise

**DOI:** 10.2471/BLT.15.020415

**Published:** 2015-04-01

## Abstract

New rapid diagnostic tests for Ebola may help Guinea, Liberia and Sierra Leone reach zero cases and allow routine health services to resume. Fiona Fleck reports.

When the Ebola virus disease outbreak was confirmed in Guinea last year, Dr Pierre Formenty and his team working on emerging and dangerous pathogens at the World Health Organization (WHO) in Geneva called on their network of collaborating laboratories for help.

“When the outbreak started it was small, so we deployed two mobile field labs in Guinea, one in Guékédou run by the European Union Mobile Lab consortium, and the other in Conakry, run by the Pasteur Institute in Senegal,” says French virologist Formenty, who joined WHO in 1996 and has worked on virtually every Ebola outbreak since then.

“But since July last year, when the outbreak spiralled out of control, we have built up the number of mobile labs deployed in western Africa to 31,” he says, referring to the WHO Emerging and Dangerous Pathogens Laboratory Network, adding that five more laboratories are planned to meet demand for testing.

These mobile field laboratories – provided by the European Union as well as Canada, China, France, Italy, the Netherlands, Nigeria, the Russian Federation, Senegal, South Africa, the United Kingdom and the United States of America and other countries – working alongside Ebola treatment units have played an essential role in fighting the outbreak.

Not only have these laboratories done diagnostic tests to confirm whether suspected cases are positive, but they have also shared the test results with WHO, which has used these to compile – and publish online – regular updates on the outbreak.

“By following the trends of samples tested and updating the figures every day, at WHO we have been able to track and analyse the different waves of the epidemic in real time and create a database for the three countries,” Formenty says.

The diagnostic tests that have been used thus far in the outbreak use the reverse-transcriptase polymerase chain reaction (RT–PCR) method to detect the virus’s genetic material. They are highly accurate in detecting cases and ruling out non-cases.

**Figure:**
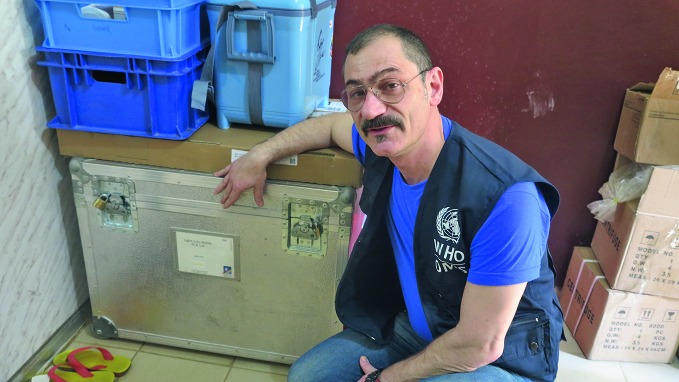
Equipping a laboratory to run Ebola tests in Conakry, Guinea

WHO/MA Heine

But there are several drawbacks to the use of RT–PCR in the current outbreak. “The tests are complex, take several hours to run and require well-equipped laboratory settings (either in previously-established laboratories or in the newly-installed mobile laboratories) and well-trained personnel,” says Francis Moussy, a biomedical engineer who leads WHO’s initiative on diagnostics innovation.

The consequences are dire.

“Some patients travel quite far to be tested and so it takes several days from the time that their symptoms appear to the time they are diagnosed with Ebola,” says Dr Arlene Chua, the diagnostics policy advisor at Médecins Sans Frontières (MSF) Access Campaign. MSF runs several Ebola treatment centres in the western Africa epidemic.

“This means that if they are indeed infected with Ebola, they could be transmitting the virus while they are travelling and waiting for tests,” she says, adding: “The faster you can diagnose Ebola, the sooner you can manage the patients and prevent further transmission from happening.”

“The faster you can diagnose Ebola, the sooner you can manage the patients and prevent further transmission from happening.”

Arlene Chua

“Rapid diagnosis is key to controlling the outbreak,” Chua says.

Last October, WHO joined forces with MSF and the Foundation for Innovative New Diagnostics (FIND) to issue a target product profile – to guide the diagnostics industry in the development of rapid point-of-care Ebola diagnostic tests for use in the outbreak.

The idea was to encourage development of accurate diagnostic tests that would be more suitable for use in countries in western Africa and that are simple and safe to use.

FIND’s chief scientific officer Mark Perkins said: “We are taking the normal process of research and development along with evaluation and implementation, which takes somewhere between two and 10 years and trying to compress this into two to 10 months.”

Last December, WHO hosted a meeting with partners FIND and MSF to discuss how to speed up the development and roll-out of rapid and safe diagnostics and laboratory systems for Ebola, and how to prepare the ground for the introduction of these new tests in the three most affected countries.

If any of these new tests prove they can accurately diagnose cases and rule out non-cases, they could play a key role in ending the epidemic and allowing routine health services to resume.

Several cartridge-based RT–PCR diagnostic products are under development. These allow the patient’s specimen to be loaded into a reagent-filled cartridge to be directly placed into a machine and do not require any additional manipulation by the user, making it possible to be used at point-of-care.

**Figure:**
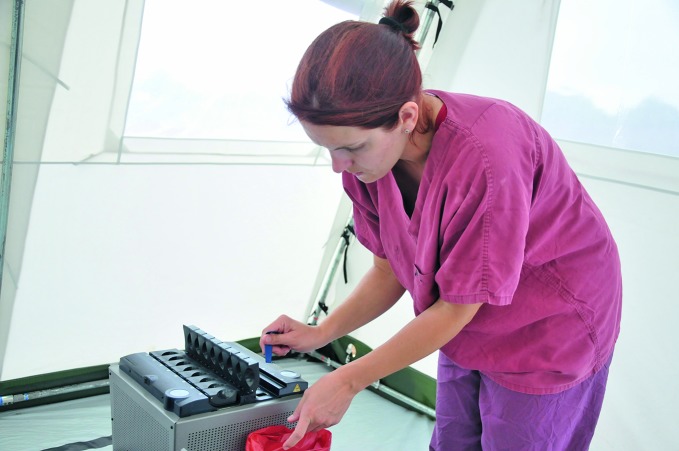
Field laboratory facilities serve an Ebola emergency medical unit in Monrovia, Liberia

WHO/A Bhatiasevi

In parallel, WHO launched a mechanism known as “emergency use assessment and listing” and invited diagnostic manufacturers that had already developed Ebola diagnostic tests to submit applications.

“The idea of the new mechanism is to provide some assurance that any newly developed diagnostic test will be of sufficient quality for use in the outbreak,” says Robyn Meurant, from the team working on the new mechanism in WHO’s essential medicines department. “Thus, any diagnostic tests assessed and deemed acceptable would be eligible for WHO procurement.”

By the end of February, 22 applications had been received.

The majority were for either RT–PCR tests – including some that could be used at point-of-care and, thus, would not have to be sent to mobile laboratories – and rapid antigen detection tests that may also be used at point-of-care.

In November 2014, the first product to successfully undergo such assessment was listed as suitable for procurement: the RealStar® Filovirus Screen RT–PCR Kit 1.0 manufactured by German company Altona Diagnostics GmbH.

Following an assessment of the ReEBOV™ Antigen Rapid Test Kit developed by US-based company Corgenix Medical Corporation, WHO announced in February that this was the first rapid test for Ebola that was eligible for procurement. Other products are also being assessed under WHO’s emergency use assessment and listing procedure.

“Most of the health clinics in the affected countries are closed,” says Formenty, “if we had a rapid, reliable and safe diagnostic test in the field, we could test suspected patients with Ebola virus disease symptoms and identify cases quickly. This would give people more confidence that patients are not infected and that these clinics are safe.”

Some promising candidates could be deployed as early as this month or next, Moussy says, but notes the challenges: “We need to train people, think about distribution for the tests, how many test kits will be needed, at which centres and who will use them.”

“We need to train people, think about distribution for the tests, how many test kits will be needed, at which centres and who will use them.”

Francis Moussy

WHO is currently developing a guide on the use of such rapid tests, but there are many challenges with their implementation.

“Many issues with rapid diagnostic tests for Ebola relate to whether the testing procedure is of sufficiently high quality to ensure accurate results,” says Meurant.

Other challenges for implementation include ensuring that health workers in remote centres wear full protective clothing while carrying out the tests, have been properly trained and have clear step-to-step instructions on all pre-test, testing and post-test procedures.

“In recent months case numbers have been dwindling and the outbreak is becoming more diffuse, with multiple tiny flare-ups across Guinea, Liberia and Sierra Leone, and that is why testing must be decentralized to these remote sites,” Formenty says.

“Whilst waiting for more sensitive, point-of-care RT–PCR tests to become available, the ReEBOV™ Antigen Rapid Test has the potential to be of good use in the investigation of a suspected outbreak in a remote site, where a cluster of cases appear to have symptoms of Ebola and a quick response is required,” Meurant says, adding:

“If several patients are diagnosed positive with the rapid test, one could start isolation and contact tracing to stamp out the flare up whilst sending the specimens for confirmation by RT–PCR. This can save lives and considerable time.”

